# Theoretical and Computational Studies of Peptides and Receptors of the Insulin Family

**DOI:** 10.3390/membranes5010048

**Published:** 2015-02-11

**Authors:** Harish Vashisth

**Affiliations:** Department of Chemical Engineering, University of New Hampshire, 33 Academic Way, Durham, NH 03824, USA; E-Mail:Harish.Vashisth@unh.edu; Tel.: +1-603-862-2483; Fax: +1-603-862-3747

**Keywords:** insulin, insulin-like growth factors, receptor tyrosine kinases, computer simulations, docking, enhanced sampling, signal transduction

## Abstract

Synergistic interactions among peptides and receptors of the insulin family are required for glucose homeostasis, normal cellular growth and development, proliferation, differentiation and other metabolic processes. The peptides of the insulin family are disulfide-linked single or dual-chain proteins, while receptors are ligand-activated transmembrane glycoproteins of the receptor tyrosine kinase (RTK) superfamily. Binding of ligands to the extracellular domains of receptors is known to initiate signaling via activation of intracellular kinase domains. While the structure of insulin has been known since 1969, recent decades have seen remarkable progress on the structural biology of apo and liganded receptor fragments. Here, we review how this useful structural information (on ligands and receptors) has enabled large-scale atomically-resolved simulations to elucidate the conformational dynamics of these biomolecules. Particularly, applications of molecular dynamics (MD) and Monte Carlo (MC) simulation methods are discussed in various contexts, including studies of isolated ligands, apo-receptors, ligand/receptor complexes and intracellular kinase domains. The review concludes with a brief overview and future outlook for modeling and computational studies in this family of proteins.

## 1. Introduction

### 1.1. Brief Historical Account

Insulin, a peptide hormone described as an “enduring medical miracle” [1] and the “protein of the 20th century” [2], was discovered in 1922 by the team of Frederick Banting, Charles Best, James Collip and John Macleod [3,4,5,6]. Insulin is secreted by pancreatic *β* cells [7] and is primarily responsible for glucose homeostasis in higher organisms. Early successes of insulin administration in dogs and then in humans [8,9] led to the large-scale production of insulin [10] and discoveries of its prolonged action [11,12]. Later, the biosynthetic pathway of insulin was discovered [13], radioimmunoassays for measuring minute circulating amounts of insulin were developed [14,15], the concept of insulin analogues was introduced [16,17,18], the physicochemical basis for rapid time-action of some insulin analogues was elucidated [19,20] and other methods of insulin delivery were explored [21,22,23,24,25].

### 1.2. Structural Biology of the Insulin Family

On the structural biology front, insulin is also considered a model protein, as it was the first protein to have its primary structure sequenced [26], followed by the determination of its three-dimensional structure [27], a discovery that has inspired extensive work on the structural studies of various insulin forms and related ligands [28,29,30,31,32,33,34,35,36,37,38,39,40,41,42]. Soon came evidence for a cell surface receptor for insulin, the insulin receptor (IR) [43], followed by its classification as a receptor tyrosine kinase (RTK) [44,45] and the determination of its primary sequence, as well as of a related homologue, type-1 insulin-like growth factor receptor (IGF1R) [46,47,48]. These findings collectively suggested that insulin shares its signaling pathways with other ligands, such as growth factors, mainly type-1 and type-2 insulin-like growth factors (IGF1 and IGF2), which exert their physiological effects via IGF1R, but can also cross-react with IR [49,50]. In fact, the mammalian insulin peptide family consists of insulin, IGF1, IGF2, seven relaxin peptides, six soluble IGF binding proteins (IGFBPs), IR, IGF1R and insulin receptor-related receptor (IRR) [51,52]. The IGFs/IGF1R system has been implicated in tumorigenesis, cancer development and progression [53,54,55,56,57,58,59,60,61], while IRR has been suggested to function as an alkali sensor [62]; we will focus here only on interactions among insulin, IGF1, IGF2, IR and IGF1R.

Although intact structures of full-length receptors remain elusive and have been listed among highly desired structures [63], the structural biology community has made consistent and steady progress in solving the structures of various parts of receptors in different forms:

(1) Intracellular kinase domains: The first crystal structures of the human IR kinase domain (IRKD) in inactive and active forms were determined in 1994 [64] and 1997 [65], respectively. Similar inactive and active structures of the IGF1R kinase domain (IGF1RKD) were later determined in 2001 [66] and 2002 [67], respectively.
(2) Apo-ectodomains: In 1998 came the first breakthrough when Colin Ward and colleagues reported the atomic crystal structure of the first three domains of IGF1R [68]. The next year, they further reported the first electron microscopy (EM) images of the human insulin receptor ectodomain and its complexes with antibody fragments [69]. After a gap of seven years, the same group reported ground-breaking discoveries on the crystal structures of the first three domains of IR [70], as well as of the IR ectodomain (IRΔ*β*) [71]. The original IR ectodomain structure (PDB Code 2DTG) was later improved (PDB Code 3LOH) to include the previously unresolved C-terminal region of the IR *α*-chain (also known as the *α*CT peptide) [72]. Consistent with small-angle X-ray scattering (SAXS) data, a homology model of the IGF1R ectodomain (IGF1RΔ*β*) based on IR crystal structures was constructed in 2009 by Whitten *et al.* [73]. During the aforementioned seven-year gap, Luo *et al.* [74] reported the quaternary structure of the insulin-IR complex based on EM images. We note that this quaternary structure has been under debate due to inconsistency with the crystal structure of the IR ectodomain [2,49,71,75].
(3) Ligand-bound ectodomains: In other major breakthroughs, an international team, comprised of Colin Ward, Michael Lawrence, Michael Weiss and colleagues, reported a series of structures of insulin bound to various constructs of the IR ectodomain in 2013 [76] and 2014 [77].
(4) Transmembrane domain: Li *et al.* [78] have recently reported a solution structure of the transmembrane domain of human IR using NMR spectroscopy.

No other experimental structures of IR or IGF1R have been reported to date (to the best of our knowledge). To gain a better understanding of the significance of these structures and structure-function relationships in the IR family, we refer interested readers to a series of earlier comprehensive reviews [2,5,49,50,52,75,79,80,81,82,83,84,85,86,87,88,89,90,91]. Additionally, the following reviews, commentary and perspective articles on the insulin family are recommended [51,92,93,94,95,96,97,98,99,100,101]. In this short review, we instead will focus on new questions raised by the outlined structures concerning the conformational dynamics of these ligands and/or receptors that have been answered using detailed theory, modeling and simulation approaches. In the following, we first briefly review key structural details and the domain nomenclature of ligands and receptors; then, we describe the modeling and simulation approaches that have been applied and, finally, summarize the applications of these methods to individual ligand/receptor systems. We conclude the review with a brief section on future outlooks and unanswered questions that can be potentially addressed using biophysical simulations.

## 2. Structural Details: Architecture and Nomenclature

### 2.1. Ligands

**Figure 1:**
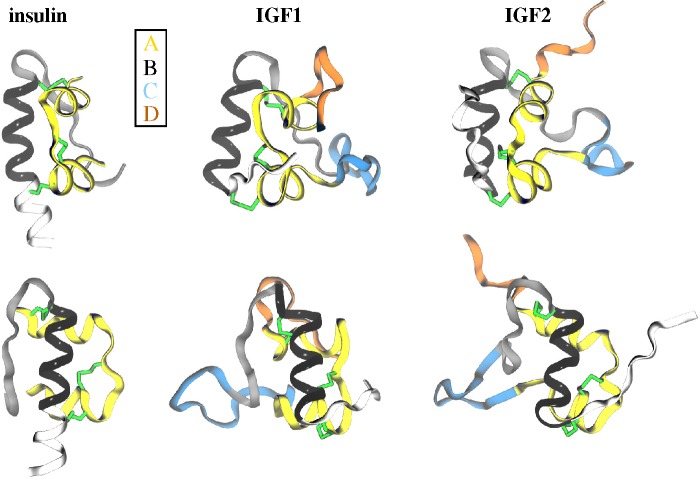
Cartoon representations of the three-dimensional folds of insulin, IGF1 and IGF2: A chains/domains are yellow; C and D domains are blue and orange; while parts of B chains/domains are shown in white (N-terminus), black (middle helix) and gray (C-terminus). Disulfide bonds are represented by greens sticks. (**top**) Side views focusing simultaneously on A and B chains/domains; (**bottom**) views focusing on the B chains/domains with other parts of ligands hidden behind.

Insulin is synthesized as a single-chain molecule of ~110 residues, also known as preproinsulin, which becomes proinsulin on immediate removal of the signal sequence and then mature insulin on cleavage of the connecting peptide by enzymes. Therefore, the final insulin monomer is composed of two chains, an A chain with 21 residues and a B chain with 30 residues. However, homologous growth factors IGF1 (70 residues) and IGF2 (67 residues) are single-chain polypeptides, each with four domains designated conventionally as B, C, A and D (from the N-terminus to the C-terminus), respectively. The A and B domains of IGFs are similar to the A and B chains of insulin, while insulin lacks the C and D domains, unlike IGFs. However, each ligand has three disulfide bonds, and IGFs are also slightly larger in size than insulin due to additional domains. The three-dimensional folds of all ligands are shown in Figure 1. Similar structural elements among ligands include two *α*-helices (yellow) in A chains/domains and a central *α*-helix (black) in the B chains/domains. Key differences are in the N- and C-termini of B chains/domains: the N-terminus of insulin B-chain can be either extended (**T**-state like IGFs) or helical (**R**-state), while the C-terminus of insulin B-chain can form a *β*-sheet, unlike the unstructured conformation seen for IGFs. The C and D domains of IGFs, absent in mature insulin, are devoid of well-defined secondary structure elements.

**Figure 2:**
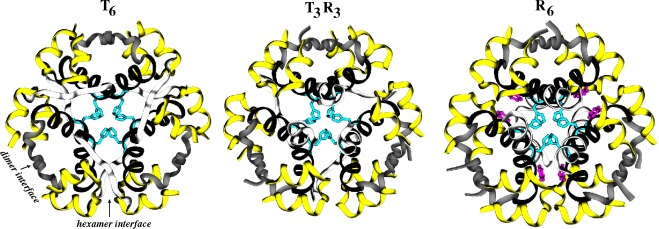
Three allosteric forms of insulin hexamers. **T**_6_, **T**_3_**R**_3_ and **R**_6_ insulin hexamers are shown in cartoon representations. The color scheme for the insulin monomers is the same as in Figure 1. Additionally, six zinc-coordinating histidine residues from six B chains (cyan) and six phenols (magenta; **R**_6_ only) are shown in stick representations. Disulfide bonds are omitted for clarity. Approximate locations of two key interfaces (dimer and hexamer-forming) are also marked (**left**).

The unique features present in the N- and C-termini of the insulin B-chain allow the hormone to dimerize or hexamerize (in the presence of zinc or phenol) via self-assembly. Such insulin hexamers can exist in a dynamic equilibrium between three allosteric states, known as **T**_6_,**T**_3_**R**_3_ and **R**_6_ [28,29,30,31,32,33,34,35,36,37], that can be shifted to **R**_6_ only by phenolic species [102,103,104]. However, one can achieve the **T**_3_**R**_3_ state by phenolic species or concentrated anionic medium or both. Six hydrophobic pockets exist for phenolic ligands in **R**_6_ hexamers, but not in **T**_6_ hexamers. The overall arrangement of insulin monomers in three hexameric allosteric states is shown in Figure 2. No structural evidence exists for the oligomerization or conformational change in IGFs, but it has been suggested that the N-terminus of IGF1 may undergo a conformational change that affects its receptor binding affinity [105].

### 2.2. Receptors

Insulin and insulin-like growth factors initiate signaling by binding to their cognate cell-surface receptors, namely IR and IGF1R; although both receptors can bind insulin, IGF1 and IGF2 with differing affinities. The sequences of receptor precursors contain ~1370 (IR) and ~1367 (IGF1R) residues, respectively [46,47,48]. Each receptor is a ~300–350-kDa protein having two subunits, each with two chains (*α* and *β*). The *α*-chains are entirely extracellular, and the *β*-chains have parts on the extracellular side, in the membrane and on the intracellular side. The formation of *α*_2_*−β*_2_ mature receptors requires dimerization, glycosylation [106,107,108] and proteolytic processing of precursors. Among RTKs, the covalently-linked [109,110] homodimeric architecture of highly homologous IR and IGF1R is unique in that these receptors require domain rearrangements rather than receptor dimerization for activation [50].

**Figure 3:**
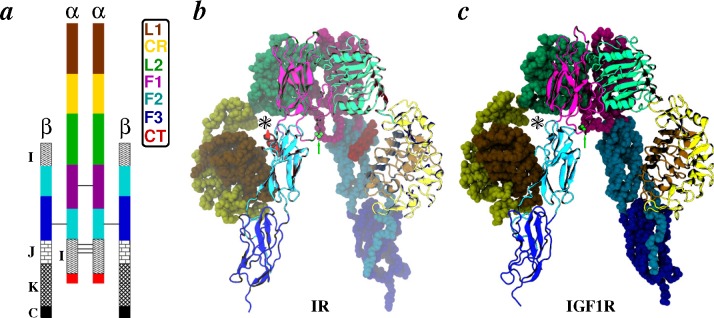
The domain organization and structures of receptor ectodomains. (**a**) A schematic of the domain organization in full-length receptors is shown. The *α*- and *β*-chains, as well as receptor domains are labeled. Labels are in the same color as the domains, except insert domains (**I**), juxtamembrane regions (**J**), kinase modules (**K**) and C-terminal tails (**C**), all of which are depicted by filled patterns; (**b,c**) Three-dimensional folds of IRΔ*β* (PDB Code 3LOH) and IGF1RΔ*β* (homology model of Whitten *et al.* [73]) are shown with domains of one subunit as space-filling, while identical domains of the other subunit are shown as cartoons. All domains are uniquely colored as in (a). One intersubunit disulfide-bond resolved at Cys^524^ in IRΔ*β* and modeled at Cys^514^ for IGF1RΔ*β* is shown in green sticks (indicated by green arrows). The *α*CT peptide is shown only for IRΔ*β*, as it was resolved [72] after publication of the homology model of IGF1RΔ*β* [73]. The location of one out of two binding pockets in each receptor ectodomain is marked by an asterisk; see Figure 4a for a side view of this binding pocket.

Each subunit in receptor homodimers starts with two leucine-rich domains (L1 and L2) separated by a cysteine-rich (CR) domain and followed by three type III fibronectin repeats (F1, F2 and F3) on the extracellular side. The C-terminus of F3 is connected to the cytoplasmic kinase modules via single-pass transmembrane domains. The structural evidence of domain organization in IR and IGF1R came from various crystal structures: the L1-CR-L2 motifs from IGF1R (PDB Code 1IGR) [68] and IR (PDB Code 2HR7) [70], and IRΔ*β* (PDB Codes 2DTG and 3LOH) [71,72]. A homology model of IGF1RΔ*β* validated using small-angle X-ray scattering (SAXS) data further confirmed the similarity in the overall topology of IR and IGF1R [73]. As depicted in Figure 3, the ectodomains of both receptors have a symmetric folded-over (∧-shaped) architecture with the following key features: (1) the L1-CR-L2 domains of one subunit and the F1-F2-F3 domains of the other subunit form one binding pocket on each side of the receptor; (2) the major contact surfaces between two subunits are at the L1-F2 interfaces near each binding pocket and at the L2-F1 interfaces near the apex of each ectodomain; (3) the *α*CT helical peptide located on the L1 surface is also part of each ligand binding pocket; and (4) the F3 domains (“legs” of receptors) are proximal to the membrane.

#### 2.2.1. Conformational Metrics of Receptors

**Figure 4:**
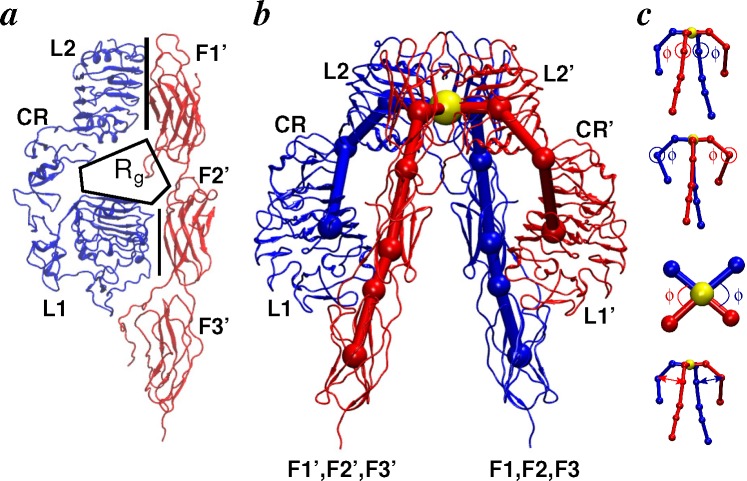
Conformational metrics of receptors. (**a**) Schematic illustration of a side view of the IR ectodomain. The binding pocket (indicated by R_g_) is formed by the L1-CR-L2 motif of one subunit (blue) and the F1-F2-F3 motif of the other subunit (red). The L2-F1′ and L1-F2′ interfaces are indicated by vertical lines; line thickness indicates higher/lower buried surface area; (**b**) Overlay of mapping points on the crystallographic conformation of the IR ectodomain. Each subunit is conceptualized as a linear chain of eight mapping points (indicated by spheres) with an additional mapping point (yellow sphere) joining both subunits at the apex. Each mapping point corresponds to either the center-of-mass of a domain or an interdomain hinge; (**c**) Hinge angles (F1-F2, L1-L2 and L2-F1) are indicated (**top**), and the interhinge distances between the L1-L2 and F1-F2 hinge points are also shown (**bottom**).

We have previously defined some quantitative geometric measures for receptor ectodomains [111,112], such as the radius of gyration (R_g_) of each binding pocket, the buried surface areas between the L1/F2 and L2/F1 domains and the interdomain hinge angles, as well as the interhinge distances based on the centers of mass (COM) of each domain. These geometric measures of ectodomains are shown in Figure 4 and suggest that: (1) the L1-L2 hinges are at ~90° in each receptor; (2) the F1-F2 hinges are at ~161° (IR) and ~166° (IGF1R); (3) the apical L2-F1 hinges are at ~82° in each receptor; (4) interhinge distances between the L1-L2 and F1-F2 hinge points are at 51 Å (IR) and 50 Å (IGF1R); (5) the R_g_ values are 20 Å (IR) and 22.5 Å (IGF1R); (6) the buried surface areas at the L1/F2 interfaces are ~900 Å^2^ (IR) and ~1300 Å^2^ (IGF1R); and (7) the buried surface areas at the L2/F1 interfaces are ~1100 Å^2^ (IR) and ~1,600 Å^2^ (IGF1R).

### 2.3. Ligand/Receptor Interactions

**Figure 5:**
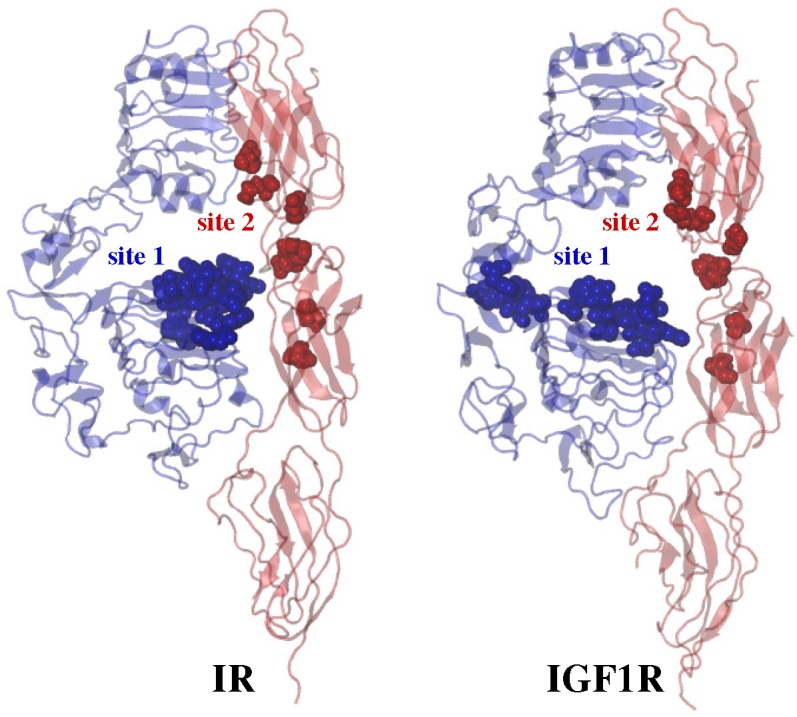
Site 1 and site 2 residues on IR and IGF1R. Schematic illustration of a side view of IR and IGF1R ectodomains indicating site 1 (blue) and site 2 (red) residues in space-filling representations; see Figure 4a for domain labels and the coloring scheme. Site 2 residues are located in the F1-F2 motif of each receptor, while site 1 residues span the L1 (IR) and L1-CR (IGF1R) domains.

A series of experimental studies on IR [2,49,75,80,85,113,114,115,116,117,118,119,120,121,122,123,124,125,126,127,128,129,130,131,132,133,134,135,136,137] and IGF1R [59,138,139,140,141,142,143,144,145,146,147,148,149,150,151,152,153,154,155,156,157,158,159,160,161,162,163,164,165,166,167,168] systems have revealed that ligands and receptors interact via two surfaces known as “site 1” and “site 2”. Both chains of insulin contribute residues to each site, and similarly, residues in different domains of IGFs are part of each site. Specifically, site 1 on each ligand is comprised of the following residues: G1, I2, V3, Q5, T8, Y19, N21 (insulin A chain); V12, Y16, F24, F25, Y26 (insulin B chain); A8, V11, F23, F24, Y31, R36, R37, V44, A62 (IGF1); and V14, Q18, F26, F28, Y27, S29, S33, V43, F48 (IGF2). Site 2 on each ligand has the following residues: S12, L13, E17 (insulin A chain); H10, E13, L17, V18 (insulin B chain); E9, D12, F16, R21, D53, L54, R56, M59, E58, Y60, K65, K68 (IGF1); and T7, L8, E12, D15, F19, L53, E57 (IGF2). For IR, the site 1 residues (D12, I13, R14, N15, Q34, L36, L37, F39, E44, F64, Y67, F89, N90, Y91, F705, E706, D707, Y708, L709, N711, V712, F714, P716 and R717) are primarily in L1/*α*CT, and the site 2 residues (K484, L552, D591, I602, K616, D620 and P621) are in loops of the F1-F2 pair; while for IGF1R, the L1-CR/*α*CT motif contains site 1 residues (D8, N11, Y28, H30, L32, L33, L56, F58, R59, W79, F90, R240, F241, E242, F251, F692, E693, N694, L696, H697, N698, I700, F701; IGF1 and/or IGF2), and loops of the F1-F2 motif neighboring each binding pocket likely contain site 2 residues (R474, L537, N577, L588, N602, L606, P607; IGF1 and/or IGF2) (Figure 5). Additionally, receptor activation and ligand binding displays allosteric properties, such as high- and low-affinity binding sites, negative cooperativity and ligand dependence of the receptor dissociation rate [84,169,170,171]. Furthermore, the receptors can bind only a single ligand molecule with high affinity and at least another one with lower affinity [169]. The stoichiometry of insulin binding to the IR ectodomain in the presence of the free CT peptide is known to be 2:2 (insulin:IR subunit) [124,126,130], and small-angle X-ray scattering studies have suggested that between one to three molecules of IGF1 can bind to IGF1RΔ*β* [73].

## 3. Modeling and Simulation Techniques

Two fundamentally different molecular simulation techniques to study complex (multi-atom) biological systems are molecular dynamics (MD) simulation and Monte Carlo (MC) simulation [172,173,174,175]. Both techniques have been used in various studies of ligands and/or receptors of the insulin family, and therefore, in the following, we briefly review the basics of MD and MC simulations.

### 3.1. Molecular Dynamics Simulations

MD simulation is an approach to compute the time evolution of individual particles (or atoms) interacting under the influence of an interatomic potential function (also known as a force-field) by numerical integration of Newton’s equations of motion [176,177,178]. The state of a system of *N* particles in a volume *V* in MD is specified by providing Cartesian coordinates of all particles and corresponding values of initial velocities. The biomolecular force-fields often explicitly account for all bonded (arising from bond-stretching, angle-bending and torsional interactions) and non-bonded forces (arising from van der Waals and electrostatic interactions). Among others, a popular time-tested interatomic potential function for proteins is CHARMM (Chemistry at Harvard Macromolecular Mechanics) [179,180].

**Figure 6:**
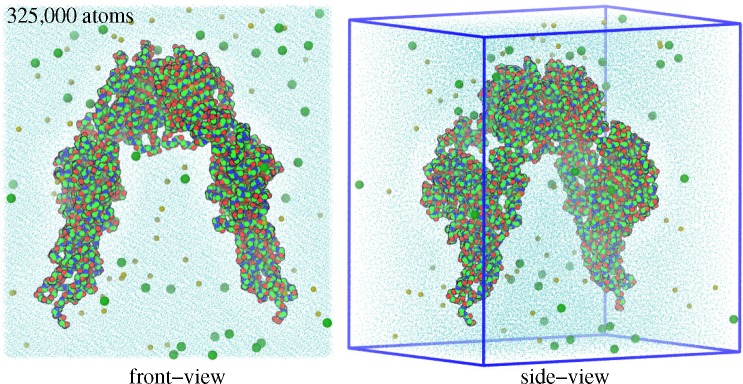
MD simulation domain. Front and side views of solvated and ionized IGF1RΔ*β* are shown. The simulation domain measures 158 × 170 × 126 Å^3^ and contains ~325,000 atoms. Protein, ion and water molecules are shown in space-filling, spherical and wireframe representations, respectively.

An MD simulation begins with an initial configuration of the biomolecule, the atomic positions for which are typically extracted from the experimental structures deposited in repositories, such as the Protein Data Bank (PDB) (www.rcsb.org). The PDB structures may contain high-energy interactions due to atomic overlaps, which need to be removed by an energy minimization procedure before beginning the dynamics. The initial configurations are further solvated with explicit water, and counterions are added for maintaining the overall charge neutrality of the system. Hence, the final simulation system may contain thousands of atoms (Figure 6), including those of protein, solvent, ions and small molecules, if any. The initial velocities of all particles are randomly assigned from a Maxwell–Boltzmann distribution at a specified temperature (often ~300 K). The temperature and pressure control in MD simulations is achieved by various schemes that implement an algorithm for a thermostat or a barostat [181,182,183,184,185]. Additionally, periodic boundary conditions are applied by replicating the central unit cell to infinity in all directions; in three dimensions, each unit cell will have 26 nearest neighbors. Many simulation packages, such as NAMD (Nanoscale Molecular Dynamics) are now freely available (for academic users) to carry out MD simulations of biomolecules [186,187]. Visualization and analysis of resulting simulation trajectories can be done with software packages, such as VMD (Visual Molecular Dynamics) [188]. The quality of the initial model, the degree of sampling and the accuracy of the force-field are a few key factors determining the success of an MD simulation [189].

### 3.2. Monte Carlo Simulations

MC simulation is a method of random sampling, which means that it can be used to generate newer configurations of a system by randomly changing the initial positions of constituent atoms. Metropolis *et al.* [190] originally proposed importance sampling using MC simulations as a way to bias the random generation of those configurations that make the most significant contribution to the desired property. Each small random move that displaces an initial configuration is known as a trial move. MC simulations are most commonly used for docking calculations in biomolecular systems by using translational and orientational displacements as trial moves. The interaction energy of selected atoms or the potential energy of the new configuration can be used as a basis to accept or reject moves using a Boltzmann factor weighting scheme. In the insulin family, MC simulations have been primarily used for docking ligands into binding pockets of receptors with trial moves comprising rigid-body translational/orientational displacements and configurational sampling of ligands/receptors from pre-equilibrated trajectories to account for biomolecular flexibility [111,112,191].

### 3.3. Enhanced Sampling and Free Energy Methods

Given the many degrees-of-freedom in biomolecules and a small numerical integration time-step (~1-fs) in MD simulations, it is often not possible to generate long enough trajectories that can capture large-scale and barrier-mediated conformational changes in biomolecules. Therefore, many new simulation techniques based on MD have been devised to increase the likelihood of the observation of such rare events. These “enhanced sampling” methods often explore phase space in reduced collective coordinates (e.g., angles, distances, *etc.*) and, thereby, are suitable for exploring long time-scale phenomena. In the following, we briefly describe two such techniques, temperature-accelerated molecular dynamics (TAMD) and the string method in collective variables (CVs), which have been jointly used to study conformational changes in the C-terminus of the insulin B chain and in the activation loop of the IR kinase domain [191,192]. For more details on enhanced sampling techniques, we refer interested readers to recent reviews [193,194].

#### 3.3.1. Temperature-Accelerated Molecular Dynamics

Temperature-accelerated molecular dynamics (TAMD) is an enhanced sampling approach to explore the physical free-energy landscape in a large set of CVs [195,196,197,198,199,200,201,202]; CVs here are functions of atomic Cartesian positions. In TAMD, additional auxiliary variables are harmonically coupled to CVs, assigned a fictitious mass and a temperature different from that of the physical system. Furthermore, slower evolution of auxiliary variables is guaranteed by using a higher Langevin friction coefficient on these variables. Due to coupling with the atomistic system, sufficiently high temperature on fictitious variables therefore leads to enhanced sampling of the physical free-energy landscape. For generating conformational changes in the C-terminus of the insulin B chain and the kinase domain loop, Cartesian coordinates of centers of mass of spatially contiguous groups of residues were used as CVs in combination with fictitious thermal energies of 5–6 kcal/mol [191,192].

#### 3.3.2. String Method in Collective Variables

Given that TAMD only explores the underlying free-energy landscape without actually reconstructing it, other techniques, such as the string method in CVs [203], are needed to quantify free-energy differences between states visited by TAMD [191,192]. By iterative refinement of an initial pathway (such as those generated via TAMD), the string method allows computation of a minimum free-energy path (MFEP). The initial “string” in this technique is a collection of discrete configurations (images) of the molecular system that can be independently simulated to get estimates of mean forces on CVs and, thereby, free-energy as a function of those CVs.

## 4. Applications

Several decades of investigations using biochemical, biophysical and structural studies (*vide supra*) have provided key insights into the structure-function relationships in the insulin family, yet the dynamics of ligands and receptors remain poorly understood at the molecular level. The main questions about ligands relate to conformational changes in the termini of the insulin B chain, interactions among insulin monomers in oligomeric (dimeric and hexameric) states, stabilization of **R**_6_ hexamers via phenolic species, the contribution of the C- and D-domain of each IGF in ligand binding, *etc*. For receptors, major questions relate to subtle conformational changes in the quaternary structures that lead to: (1) “negative-cooperativity” in ligand binding; (2) low- and high-affinity ligand-bound states; (3) differing affinities of insulin and IGFs for IR/IGF1R; and (4) activation of intracellular kinase domains. Not all of these questions have been answered so far, but a limited number of computational studies that we discuss below have shed light on certain aspects of the conformational dynamics of ligands and receptors.

### 4.1. Ligands

Three different forms of insulin (monomer, dimer and hexamer) have been studied using vacuum and explicit-solvent MD simulations so far. During the emergence of the biomolecular simulation field [204], insulin played an important role as a test system on which a force-field with explicit hydrogens was tested by Wodak *et al.* [205]. They concluded that the choice of force-field may be at least as important as including solvent molecules in the simulations of proteins. Further vacuum MD studies recommended that solvent simulations are needed to better understand the solution conformations of insulin [206]. Soon after, explicit-solvent simulations of the insulin monomer and dimer demonstrated considerable flexibility in insulin structures that was suggested to reduce the propensity of insulin to form hexamers without divalent cations [207]. Despite earlier suggestions on the importance of solvent molecules, further studies in vacuum on cross-linked insulin monomers were carried out and justified as acceptable [208]. Tidor and Karplus computed the dimerization free energy of insulin as ~*−*7.2 kcal/mol [209]. The insulin dimer was further studied using 600 ps-long explicit-solvent simulations, which showed asymmetry among insulin monomers in the dimer [210]. The insulin monomer was exposed to chemical (disulfide-reduction) and thermal (high-temperature) stress with a focus on the unfolding behavior of the B chain *α*-helix [211]. This study showed that chemical stress alone results in smaller conformational changes consistent with experimental observations. A significant explicit-solvent MD study of monomeric and dimeric insulins suggested increased flexibility in the termini of the insulin B chain, separation of the C-terminus of the insulin B chain that exposes receptor binding residues in the A chain and the role of mutation at residue B24 (Phe→Gly) in inducing greater flexibility in the C-terminus of the insulin B chain [212]. Force-induced dissociation of the insulin dimer using steered MD simulations [213] suggested that the dissociation pathway depends on the relative strength of the inter-monomer interactions across the antiparallel *β*-sheet interface [214]. Residues contributing to the stability of the insulin monomer were also probed with computational alanine scanning in the same spirit as the experimental alanine scanning mutagenesis approach [215]. The excised insulin B chain was further extensively scrutinized by two different MD studies [216,217]. Particularly, high-temperature studies demonstrated flexibility in the termini of the insulin B chain and suggested the role of Gly^B8^ as a helix-breaker residue leading to **T**-like conformations of the insulin B chain [216], while the bias-exchange metadynamics study revealed three metastable basins separated by a large free-energy barrier, including a folded-state basin with a transformed and separated C-terminus of the insulin B chain [217]. A novel simulation method, known as targeted MD (TMD), was also applied to the insulin monomer, as well as the hexamer for studying the **T**→**R** transition [218,219]. Motivated by earlier studies on protein hydration [220,221], a study on water dynamics at the dimer forming surface (DFS) and hexamer forming surface (HFS) of insulin revealed that more structured water molecules with higher residence times were present at HFS in comparison to those at DFS [222].

**Figure 7:**
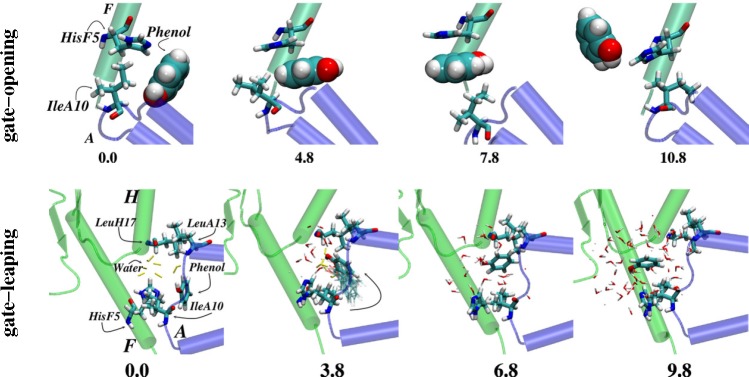
Gate-opening *vs.* gate-leaping mechanisms. Top-view and side-view snapshots for two different phenol dissociation mechanisms, gate-opening and gate-leaping, respectively. The numbers at the bottom of the panels show distances along the dissociation reaction coordinates. Additionally, two key gatekeeper residues, His^F5^ (B chain) and Ile^A10^ (A chain), are shown in sticks and labeled. Panels adapted with permission from [223].

Taking the insulin hexamer as an example for understanding the binding/dissociation of small molecules, two different studies [223,224] have attempted to explore phenol dissociation pathways from hydrophobic binding pockets of the **R**_6_ insulin hexamer (see Figure 2). Swegat *et al.* [224] found one dissociation mechanism for a phenolic ligand, while Vashisth and Abrams [223] reported multiple phenol binding/unbinding routes. The latter study computed the potentials of mean force (PMFs) for three-different phenol dissociation pathways and found two competing mechanisms namely “gate-opening” and “gate-leaping” (Figure 7). Although insulin has been investigated in many studies described above, no detailed molecular simulation studies on IGFs have been reported so far (to the best of our knowledge).

### 4.2. Receptors

Vashisth and Abrams have carried out three different simulation studies on apo and ligand-bound IRΔ*β* and IGF1RΔ*β* [111,112,191] and one study on the insulin receptor kinase domain [192]. Additionally, a new structural model of the insulin/IR complex based upon an earlier structural model [191] has been reported [225]. In the following, we describe key findings, as well as the limitations of these studies.

#### 4.2.1. Apo Ectodomains

Earlier experimental work has suggested the presence of low- and high-affinity ligand binding sites and/or “negative-cooperativity” in IR and IGF1R [170,171]. These observations were initially rationalized by Schäffer [226] in a cross-linking model in which a single ligand molecule can bridge two distinct binding sites on receptors. In the same year, De Meyts [84] proposed a better model by postulating that receptor dimers would have internal symmetry, such that site 1 on one subunit is positioned near site 2 of the other subunit (later found to be true), thereby allowing high-affinity ligand binding in one pocket and low-affinity in the other. Based on structural evidence, McKern *et al.* [71] proposed a “see-saw” mechanism of negative-cooperativity consistent with De Meyts’ model [2,49,84]. In this mechanism, the receptor ectodomain can rock back and forth between symmetry-inverted states driven by insulin binding. However, the most recent “harmonic-oscillator” model by De Meyts and colleagues [169] suggests that small thermal energies can drive the apo-ectodomains of receptors between symmetry-inverted asymmetric states potentially capable of binding ligands. Large-scale atomistic simulations of receptor ectodomains have revealed that both IRΔ*β* and IGF1RΔ*β* exhibit spontaneous asymmetric mechanisms of flexibility that directly provide evidence in favor of the harmonic-oscillator model. Such asymmetry can also be indirectly inferred from ectodomain structures, because intact crystal conformations of ligands cannot be docked [70,71] in binding pockets of receptors without significant steric overlaps. As described below, the flexibility and asymmetry in apo-ectodomains is observed at different levels in MD simulations [111,112].

Intradomain flexibility analyses from simulations suggest that: (1) the overall fold of all domains is relatively preserved in solution; (2) the CR domain and fibronectin repeats are intrinsically more flexible than L1 and L2; and (3) the N-terminal loops of CR in IR (junction of CR and L1) and the C-terminal loops of CR in IGF1R (junction of CR and L2) show increased flexibility. Interdomain and intersubunit flexibility in ectodomains can be evaluated by various geometric measures (see Section 2.2.1 and Figure 4) that characterize the relative movement of domains, the size of binding pockets and the integrity of domain interfaces. These measures have collectively suggested that closing of the F1-F2 hinge on one side of the receptor dimer moves the hinge point away from the nearest L1-L2 hinge, such that the associated L2/F1 interface opens slightly (manifest also as an increase in the apical L2-F1 hinge-angle). This in combination with the closing of the L1-L2 hinge further leads to one binding pocket being more open than the other. Therefore, the receptor dimer is locked in a state where the apical L2-F1 hinge angle, as well as the L2/F1 interface on the closed-pocket side of dimer is more intact than the open-pocket side. A large enough fluctuation can then drive the structural transition in the opposite direction, given that the F1-F2 hinge on the closed side also experiences fluctuation, leading to its movement away from the L1-L2 hinge. This way, random thermal fluctuations in interdomain hinge angles can lead to spontaneous asymmetry in the intersubunit interfaces, as well as binding pockets. This would also mean that the intersubunit interfaces (L1/F2 and L2/F1) in symmetric receptor dimers (Figure 3) are not at their optimum strength. Interestingly, independent simulations of ectodomains also found that each pocket has equal probability of opening/closing, because the same pocket could be observed at least once either in the open or closed state. Moreover, it is observed that the change in size of each binding pocket is slower than that of the interfacial areas, but matches the rate of change of hinges. Importantly, these interdomain flexibility mechanisms validate the prediction of McKern *et al.* [71] that domain movements potentially occur at the CR-L2, L2-F1 and F1-F2 junctions.

Given that the F1-F2 hinges can spontaneously close, thereby leading to asymmetric features in receptors, it was speculated that the symmetric crystallization of apo-IRΔ*β* likely occurred due to the binding of antibody fragments, in particular 83–14, which binds specifically to F1 near the F1-F2 hinge [111]. This is also consistent with the finding that 83-14 binding to IR inhibits insulin binding [227], likely because it prevents closing of the F1-F2 hinge angle, which is required to create an open pocket for insulin binding. Asymmetry in receptors was further validated by the fact that solution conformational ensembles of apo-IGF1RΔ*β* probed by SAXS experiments [73] and MD simulations [112] matched well. Simulations of apo-IRΔ*β* showed that asymmetry can also exist in various salt bridges located in the intersubunit interfaces [111]: Lys^460^ can participate in an intradomain salt bridge with Asp^464^ or in an interdomain salt bridge with Asp^574^. If the intersubunit salt bridge helps the tightening of the L2/F1 interface while the intrasubunit bridge frustrates the interface, this would suggest a crucial role for Lys^460^, consistent with mutational studies [228].

#### 4.2.2. Ligand/Receptor Complexes

**Figure 8:**
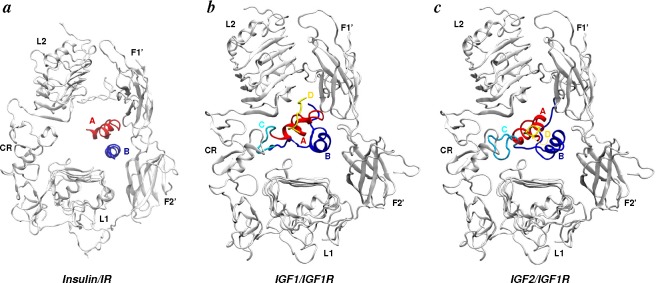
Docked conformations of insulin and IGFs. Docked views of each ligand in the relatively open binding pocket (formed by the L1-CR-L2 motif of one subunit and the F1-F2 motif of the other subunit) are shown. Both chains of insulin and four domains of each IGF are distinctly colored and labeled. (**a**) Based on the ligand/receptor complex reported in [225]; (**b,c**) based on ligand/receptor complexes reported in [112]; (b) and (c) adapted with permission from [112].

A major consequence of asymmetry in the binding pockets is that the intact ligands (insulin/IGFs) could be successfully docked in the open pockets (Figure 8) of their cognate receptors (but not in the closed pockets). All-atom structural models of ligand/receptor complexes [111,112,191,225] constructed using MC-docking calculations and MD-equilibrated thereafter were the first physically plausible complex structures based on which many experimental observations could be rationalized. The ligand/receptor complexes showed that a single ligand in the binding pocket can simultaneously contact site 1 and site 2 residues on the receptor. This is consistent with experimental observations that ligands can cross-link receptor subunits [229,230], because in all-atom structural models, each ligand contacts at least one domain of the L1-CR-L2 motif of one subunit and the F1-F2 motif of the other subunit (Figure 8). The structural models also predicted many site 2 contacts between ligands and receptors out of which site 2 residues on IR appear consistent with bioinformatics analysis [137] and mutagenesis work [131], and site 2 residues of IGF1R [112] remain testable predictions. Specifically, insulin residues (1) A12, A13, A14, A15, A17 were observed in contact with F1 residues R554, G555, L556, K557 and Y562, (2) A10 and B18 were observed in contact with F1 residues Y507 and K484, respectively; and (3) B10 and B13 were observed in the vicinity of F2 residues S596, L599, D620 and P621 [225]. Earlier structural models of insulin/IR complexes without CT peptide [111] suggested sustained contacts between insulin residues A13, A17 and B17 and receptor residues K484 and L552 (F1). A mutagenesis study by Whittaker *et al.* [131] listed K484 and L552 among the five most important residues for insulin binding in the F1-F2 motif of IR. For site 2 contacts in the IGFs/IGF1R system, all-atom structural models [112] have suggested contacts of ligand residues E9, D12, F16, D53, L54 and E58 (IGF1) and E12, D15, F19, L53 and L57 (IGF2) with IGF1R residues N473, R474, Y496, W519, N520, M521, V522, D523, L526, P528, L537, L538, H539, G540, L541, K542, Y547, V580, S582, I583, L585, L606, P607 and N608. Importantly, all IGF1/2 residues listed above were suggested as site 2 contacts by two major mutagenesis efforts [159,160].

It is known that insulin can bind only with low affinity to IRΔ*β* unless *β*-subunits are membrane-embedded or fused to dimerizing proteins [166,231,232], while IGFs can bind with high affinity to both free or leg-restricted IGF1RΔ*β* [233]. This observation in combination with studies on hybrid IGF1R/IR chimeras [136,234] possessing high affinity for IGFs has led to the possibility that the increased affinity of IGFs may be due to the presence of two additional domains (C and D), unlike insulin (Figure 1). Simulations provided strong evidence in favor of this view, because extensive additional contacts between the C- and D-domains of IGFs and the loops of the CR and L2 domains were observed [112]. Specifically, in all-atom structural models, R36 and R37 (IGF1) interact with IGF1R residues E242, E264 and E276, and IGF2 residues R37 and R38 interact with IGF1R residues E264 and E276. Furthermore, a stable salt-bridge between R37 (IGF1) and E264 (IGF1R) was observed in simulations [112]. These contacts were further consistent with earlier studies that probed the importance of additional domains in IGFs’ binding [138,141,143,148,153,167,168,235,236,237,238] and suggested electrostatic complementarity between the C-domain and a CR loop (253–266) of IGF1R [70,239]. Another important issue related to ligand recognition by receptors is the interplay of a tandem hormone binding element, the *α*CT peptide (Figure 3), and the B chain of insulin or B/C domains of IGFs. Several photo-crosslinking and mutational experiments in combination with observations on insulin mimetic peptides [72,119,128,240,241,242,243,244,245] have suggested that *α*CT is potentially mobile and may be displaced upon ligand binding. Thermodynamic calculations on all-atom insulin/IRΔ*β* models constructed with *α*CT in its crystallographic position and a displaced position [191] strongly suggested the displacement of *α*CT on insulin binding, also finally confirmed in crystal structures [76,77]. The displaced-*α*CT models [191,225] also suggested a previously known conformational change in the C-terminus of the insulin B chain [246,247,248,249] that exposes (to site 1 on IR) residues hidden in the hydrophobic core of insulin. Importantly, the comparison between insulin-bound and apo crystal structures of IR have suggested that hormone conformation in insulin-bound structures cannot be accommodated in the apo-IR ectodomain, due to the steric overlap [76,91] of insulin with the F1-F2 motif. Therefore, the displacement of the F1-F2 pair has been suggested upon insulin binding [91]. A recent all-atom structural model of the insulin/IRΔ*β* complex [225] has provided evidence in support of this view, because closing of the F1-F2 hinge leads to a displaced conformation of the F1-F2 motif that facilitates insulin binding without steric overlap.

#### 4.2.3. Intracellular Kinase Domains

As pointed out earlier, binding of ligands to receptor ectodomains leads to the activation of intracellular kinase domains. Although it is not well understood at the structural level how a ligand transmits the signal across the membrane, it is known that the activation of kinase modules in receptors occurs via *trans*-autophosphorylation of three tyrosine residues situated in the activation loop (A-loop) [250,251].

**Figure 9:**
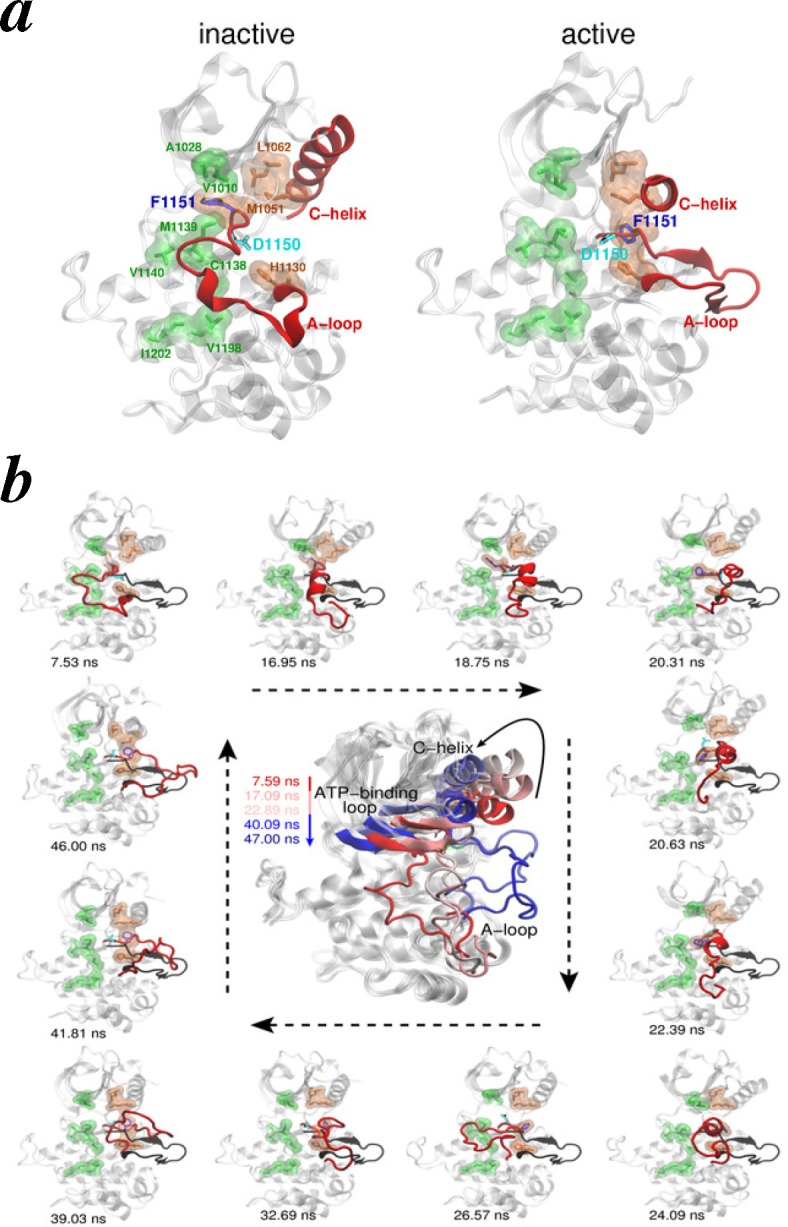
Kinase conformational change. (**a**) Key structural differences in the inactive (PBD Code 1IRK) and active kinase (PDB Code 1IR3) crystal structures are highlighted and labeled. The A-loop and *α*C-helix are shown as red cartoons, and residues of R-spine and C-spine are shown as brown and green sticks/surfaces, respectively, and labeled; (**b**) Snapshots of the kinase domain are depicted during temperature-accelerated molecular dynamics (TAMD)-generated conformational change in the A-loop. Conformation of the A-loop in the active crystal structure is also overlayed (black cartoon). Panels adapted with permission from [192].

Many structural studies on IR and IGF1R have revealed that during activation, a large conformational change involving displacement (by ~20 Å) of the A-loop occurs [64,65,66,67]. The conformational change in the A-loop is further coupled with rotation (by ~30°) of the *α*-helix in the N-lobe and the flip of an Asp(D)/Phe(F) pair in the conserved Asp-Phe-Gly (DFG) motif. Furthermore, these structural changes result in the proper placement of a network of residues collectively known as the regulatory (R) and catalytic (C) spine [252,253,254] (Figure 9a). Molecular simulations have previously provided insights into the structural transitions underlying the activation of other kinases [255,256,257,258,259].

Similarly, Vashisth and Abrams studied the conformational change in the A-loop of the IR kinase domain using TAMD simulations and the string method in CVs (see Section 3.3 for details on the methods) [192]. TAMD simulations in this work showed consistent folding of the A-loop into helical intermediate conformations that result in the flip of the DFG-motif, primarily due to changes in backbone dihedrals (Figure 9b). Detailed free-energy calculations further supported these conclusions, as the metastable helical conformations predicted by TAMD were observed to exist along the characterized minimum free energy path (MFEP). The analysis of the structural changes also revealed that the R-spine can be dynamically assembled or disassembled via the DFG-flip or the rotation of the *α*-helix in the N-lobe. In this study, an isolated kinase domain was studied in absence of ATP, and therefore, it was unclear how inter-kinase contacts or the presence of nucleotide may affect the activation pathway.

### 4.3. Limitations of Modeling Studies

Though molecular simulations have provided significant insights into the dynamics of the ligands and receptors of the insulin family, certain limitations of these modeling studies bear mentioning. Some of these include the absence of unresolved residues in the insert domain (ID) and the absence of Fab/Mab antibody fragments, as well as glycans. Moreover, simulations of IGF1R were based on a homology model that did not include *α*CT, an essential ligand binding element. The missing structural elements therefore make it difficult to attribute models to low-affinity or high-affinity complexes. The models also suggest that a bound ligand on one side of the receptor dimer may help in the opening of the binding pocket on the unoccupied side, which may allow the binding of at least another ligand. However, this has not been directly demonstrated yet, and therefore, suggestions about the stoichiometry in ligand/receptor binding may be speculative. Additionally, the reasons for the discrepancies between the theoretical *vs.* experimental scattering profiles of the solution ensembles of the IGF1/IGF1RΔ*β* complex were not apparent [112]. It is also important to point out that none of the aforementioned simulation studies of the receptors included the membrane environment, and hence, it is not obvious how the conformational changes are conveyed to the intracellular kinase domains. Lastly, due to large system sizes, simulations only explored the conformational dynamics under a ~100-ns timescale, and therefore, the conformational changes occurring on longer timescales, if any, may not have been captured in these trajectories.

## 5. Outlook

**Figure 10:**
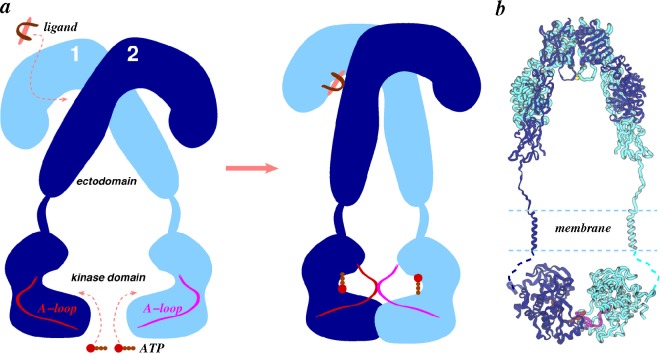
Schematics of the *holo*-receptor. Ligand-triggered compaction of the entire complex leading to the movement of intracellular kinase domains is shown (**a**), and a schematic of the entire receptor architecture constructed from known crystal structures of the IR ectodomain and the kinase domain is shown (**b**). Transmembrane helices in (**b**) were modeled. The orientation, as well as placement of the kinase dimer, are purely speculative. Missing juxtamembrane regions are shown with dashed lines, and the C-terminal tails are not shown.

In this work, we have reviewed applications of various molecular simulation techniques to ligands and receptors of the insulin family. We have focused on key questions relating to the conformational dynamics of ligands and receptors that can be suitably probed with large-scale simulations. Particularly, simulations have highlighted plasticity in ligands, as well as receptors that is potentially exploited in their productive binding. All-atom models of complexes suggest ligand cross-linking of receptor subunits and hint at the determinants of ligand specificity for their cognate receptors, an example of which is the interaction between the C-domain of IGFs with loops in the CR domain of IGF1R. Though not mentioned in the discussion above, analyses of the flexibility of membrane-proximal domains of receptors in apo- and ligand-bound forms suggest (Table S2 in [191]) that soluble apo-ectodomains display an equal probability of the opening/closing of receptor legs, but binding of ligands seems to stabilize the legs of soluble ectodomains, such that the distances between legs either decrease by ~3–5 Å or stay near values in symmetric structures. One can therefore speculate that a decrease in the distance of receptor legs can be further stabilized in the presence of membrane anchors. Interestingly, it has been observed earlier that insulin binding to membrane-anchored IR leads to compaction of the entire complex [260], as schematically shown in Figure 10a. However, two recent studies [261,262] have suggested different mechanisms [263] of IR and IGF1R activation. Kavran *et al.* [261] probed IGF1 binding to IGF1R and showed that ligand binding disrupts the L1-F2′ interface (see Figure 4a), leads to dimerization of transmembrane helices and *trans*-phosphorylation in kinase domains. Lee *et al.* [262] have shown in the insulin/IR system that constitutively dimerized transmembrane helices instead dissociate from one another on activation. These studies highlight the importance of further understanding helix dimerization/dissociation, juxtamembrane regions and other steric constraints in receptor activation. To test the role of membrane anchors, future simulation studies of receptors in the presence of membrane are needed. With the availability of structural data on ligands, receptor ectodomains, transmembrane domains and kinase domains, an ambitious goal for future model building studies is to construct a testable all-atom model of the *holo*-receptor (Figure 10b).
